# New Species of *Kronosvirus* Bacteriophages That Infect *Caulobacter* Strain CBR1

**DOI:** 10.3390/v18040437

**Published:** 2026-04-05

**Authors:** Tannaz Mohammadi, Austin Millwood, Maherah Shaik, Bert Ely

**Affiliations:** Department of Biological Sciences, University of South Carolina, Columbia, SC 29208, USA; tannaz@email.sc.edu (T.M.); austinmillwood@charter.net (A.M.); mshai48@uic.edu (M.S.)

**Keywords:** *Caulobacter*, bacteriophage, genome, phage speciation

## Abstract

*Caulobacter segnis* strain CBR1 enhances the germination rate and total biomass of *Arabidopsis* plants. Since bacteriophages are usually present in the rhizosphere, we isolated three additional bacteriophages, designated TMCBR2, TMCBR3, and W2, that can replicate using CBR1 as the host strain. The genome sizes and morphologies of the new phages were similar to those of the previously isolated Kronos phage, and when we compared the nucleotide sequence of these new phage genomes, we found only minor differences among the four phage genomes. Pairwise sequence comparisons indicated that the phage genomes should be grouped into three separate species within *Kronosvirus* genus. Interestingly, we found that most of the genomic variation occurred among genes of unknown function within a 10 kb region of the 42 kb genomes with little variation in the remaining 32 kb which contains the genes known to be important for phage propagation.

## 1. Introduction

*Caulobacter vibrioides* has long served as a key model organism for studying bacterial cell cycle regulation, intracellular organization, and cellular differentiation. Members of the genus *Caulobacter* are stalked bacteria that undergo asymmetric division, generating an immature daughter cell at each cell division [1,2]. The immature form, known as a swarmer cell, has a single flagellum located at one pole of the cell and is motile. Therefore, it can swim away from the parent stalked cell and not compete with it for limited nutrients. To mature, the swarmer cell must shed its flagellum and replace it with a stalk before it can replicate its DNA and divide. Although *Caulobacter vibrioides* is widely used as a model organism, working with it also comes with certain biological, ecological, and research-related challenges. For instance, because it naturally thrives in low-nutrient environments, its cell-cycle regulation, morphogenesis, and overall survival can change dramatically under starvation or non-optimal laboratory conditions [3]. Additionally, in environmental settings, *Caulobacter* strains can contribute to the accumulation of biofilms on surfaces, potentially leading to biofouling in water systems [4]. These ecological characteristics underscore the value of isolating phages that specifically target *Caulobacter*, both for controlling unwanted biofilms [5] and for advancing research on host–phage interactions.

Early studies of bacteriophages capable of infecting *C. vibrioides* strain CB15 resulted in a wide variety of phage phenotypes [6,7,8,9]. However, all our previous phages were lost during a freezer meltdown. Recently, our laboratory has been investigating the interactions between bacteria and bacteriophages in plant microbiomes. We have isolated and characterized diverse *Caulobacter* and non-*Caulobacter* bacterial strains from soil and plant roots [10], as well as isolating phages that exist as episomal pseudolysogens across a broad range of soil bacteria [11]. Remarkably, all 34 of these phages are jumbo phages with an elongated head and belong to the *Dolichocephalovirinae* subfamily [11]. In addition, we showed that most previously characterized *Dolichocephalovirinae* phages, including the original CbK phage [6] and more recent isolates [8,12] (unpublished results), also had a broad host range. In contrast, a lytic phage designated Kronos had a very narrow host range, infecting only the *C. segnis* CBR1 strain [13]. Kronos is smaller than the *Dolichocephalovirinae* phages with a 42 kb genome, and it has a straight noncontractile tail and an icosahedral head.

Since CBR1 can stimulate the growth of *Arabidopsis* [14], we wanted to isolate additional bacteriophages that can infect it so that we could investigate phage–host interactions that might impact the ability of CBR1 to stimulate plant growth. In this paper, we describe three additional bacteriophages that can infect CBR1. Unlike our previous *C. crescentus* phage isolations which yielded many kinds of phages [8], all of these phages were closely related to Kronos with the same morphology, size, and host range.

## 2. Materials and Methods

### 2.1. Bacteriophage Isolation and Growth

All bacterial cultures, including *C. segnis* CBR1, were grown in PYE broth at 33 °C [15]. Five water samples from three locations in North and two in South Carolina, collected by Louis Berrios in December 2019, were available so we checked them for the presence of phages that could infect CBR1. The water samples were refrigerated until they were used. To enrich for phages capable of infecting *C. segnis* CBR1, 10 mL of each water sample was processed according to the method described by Nguyen et al. [16] with phage-free water serving as a negative control on the plates containing the CBR1 host bacteria. If lysis was observed, serial dilutions of the processed water sample were prepared and mixed with 100 μL of an overnight CBR1 culture in 3.5 mL of PYE soft agar to allow for plaque formation. After individual plaques were obtained from the serial dilutions, a well-isolated plaque was purified twice by single plaque isolation before being grown by confluent lysis on PYE plates. Once confluent lysis was obtained, 6 mL of sterile PYE broth was added to the plate and refrigerated overnight to allow the phage particles to diffuse into the liquid layer. Subsequently, the liquid layer was decanted into a screw cap test tube and chloroform was added and shaken to kill any residual bacteria. Typically, this procedure yielded a high titer lysate (10^9^–10^10^ pfu/mL).

### 2.2. Host Range Analysis

The host range of each additional bacteriophage was determined by conducting both spot testing and plaque assays using various *Caulobacter* species. The tested strains included *C. segnis* strains CBR1 [13], TK0059 [17], and RBW21 [10] and *C. vibrioides* strains CB15, CB2, and CB13 [18]. To test phage sensitivity, 10 μL of each phage lysate was spotted on a PYE plate overlayed with 3.5 mL of PYE semi-solid medium (PYE plus 0.3 g agar) containing 100 μL of an overnight culture of the test strain. After overnight incubation, a clear zone was observed if the bacteriophage was able to infect that strain of *Caulobacter*. If a clear zone was observed, plating efficiency was tested by plating a series of hundred-fold dilutions on semi-solid medium containing the host bacterium to observe individual plaques.

### 2.3. Lysogen Detection

Bacteria from the centers of phage plaques were streaked on PYE plates and incubated for 40 h at 33 °C to obtain single colonies. The resulting colonies were transferred to a sterile piece of velveteen and then onto a PYE plate containing 100 μL of an overnight culture of CBR1 bacterial host in a 3 mL soft agar overlay on a second PYE plate. After overnight incubation, lysogenic colonies were identified by the presence of a halo of lysis in the indicator bacteria. This lysis halo indicates that phages capable of propagating on the indicator bacteria were present in the colony. In contrast, the absence of a halo indicated that phages were not present in that colony. To confirm lysogeny, the presumptive lysogenic colonies were restreaked and then printed on the host strain a second time. If all colonies exhibited a halo of lysis, then each bacterium that initiates an isolated colony must be associated with a phage and lysogeny is confirmed.

### 2.4. Electron Microscopy

The structural morphology of each bacteriophage was observed using transmission electron microscopy on phage samples stained with uranyl acetate. A 100 μL aliquot of a phage lysate was mixed with 100 μL of a log phase culture of CBR1 for 30 min at 30 °C. The mixture was then centrifuged and resuspended in 100 μL of autoclaved, purified water. A total of 20 μL of the resulting mixture was combined with 20 μL of 2% uranyl acetate, and a formvar/carbon 300 mesh copper grid was floated on the mixture for ~30 s. Free and adsorbed phages were then observed in a JEOL 200CX transmission electron microscope.

### 2.5. Genome Sequence Analysis and Assembly

The genomic DNA of each phage was isolated from high-titer phage lysates using a QIAamp DNA Blood Mini Kit (catalog number 51104, Qiagen, Germantown, MD, USA) according to the manufacturer’s instructions. The genomic DNA was sent to the University of Maryland genome sequencing center to determine the nucleotide sequences of the phage DNA genomes using HiFi PacBio sequencing technology to a read depth of at least 100X. Complete genomes were assembled by the genome facility using their standard procedures. The resulting genome sequences of the TMCBR2, TMCBR3, TMCBR4, and W2 phages were annotated using both the Rapid Annotation using Subsystem Technology (RAST) [19] and Pharoka [20] using the default parameters. The annotated genome sequences were compared using the Mauve [21] and Artemis [22] programs to visualize differences in gene content and gene sequence variation that was present among the phage genomes. Pairwise comparisons of the phage genomes were performed using the VIRIDIC [23], DNASTAR [DNASTAR, Madison, WI, USA], and DiGAlign (Ver. 2) programs [24]. NCBI accession numbers for the five phage genomes are Kronos, MH884648; TMCBR2, OQ269668; TMCBR3, OQ330849; W2, OQ330851.

## 3. Results

### 3.1. Phage Isolation

To isolate additional bacteriophages that could infect *Caulobacter segnis* strain CBR1, we tested lake and stream water samples from North Carolina and northern South Carolina. Four of the five water samples tested contained bacteriophages that could infect the CBR1 host strain. No phage plaques were observed with the CBR1 control or the W1SC water sample.

**Figure 1:**
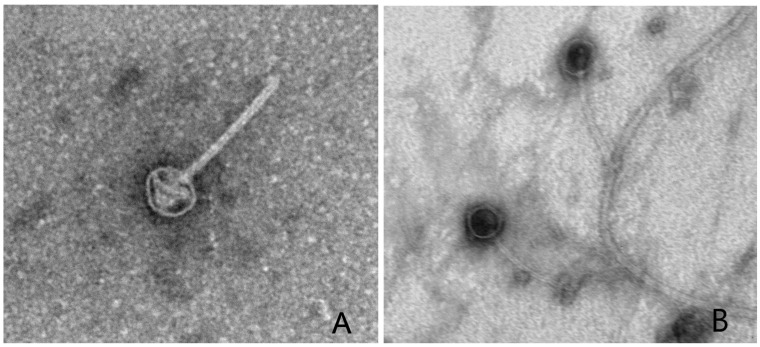
Transmission electron micrographs of representative phage particles. (**A**) TMCBR3 and (**B**) W2.

Purified isolates of these four phages were designated TMCBR2 (from sample W1NC), TMCBR3 (from sample W2NC), TMCBR4 (from sample W2SC), and W2 (from sample W2NC). However, since TMCBR4 and W2 had identical genome sequences, they were considered independent isolates of the same phage. All of the new phages have the same limited host range as Kronos, infecting only the original CBR1 host. Also, like Kronos, none of the new phages could establish lysogeny. Transmission electron microscopy (Figure 1) showed that all four phages have a 150 nm noncontractile tail and a 50 nm icosahedral head similar to those of the previously described Kronos phage [17]. Also, like Kronos, phage particles of each of the newly isolated phages seemed to attach at random locations on the surface of the CBR1 host cells.

### 3.2. Genome Comparisons

The genome sizes ranged from 42 to 43 kb with a GC content that was approximately 2% lower than that of the 68.2% GC content of the CBR1 host strain (Table 1). The annotated genomes contained 58–62 protein-coding genes and no tRNA genes (Supplementary Table S1). Most of the genes in these phage genomes are transcribed from left to right (using the orientations of the genomes in the NCBI database) with genes that code for the head and tail components located in the first half of the genome. These structural genes are followed by a set of 19 genes that are transcribed in the opposite direction and code for proteins of unknown function. The remaining genes are transcribed in the same direction as the genes for the structural proteins, but they also code for proteins of unknown function.

**Figure 2:**
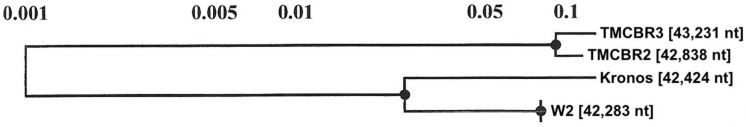
A phylogenetic tree of the *Kronosvirus* genomes.

To determine how closely the three new phage genomes were related to Kronos and to each other, pairs of genomes were compared using the VIRIDIC program [23] in all pairwise combinations. The resulting data indicated that the genomes of TMCBR2 and TMCBR3 were closely related to each other (96.1% nucleotide identity), but they were less than 95% identical to the Kronos and W2 genomes (Table 2). Also, the genomes of the W2 isolate were more distantly related to the TMCBR2, TMCBR3, and Kronos genomes (less than 95% identical). Recently, Turner et al. [25] published guidelines that stated that members of the same bacteriophage species should have at least 95% genome nucleotide identities in pairwise comparisons and that members of the same genus should have at least 70% genome nucleotide identity in pairwise comparisons. Therefore, based on these criteria, we concluded that all five genomes are similar enough to be considered members of the *Caudoviricetes Kronosvirus* bacteriophage genus [26]. However, based on the VIRIDIC comparisons, they should be classified as three different species of the same genus (Table 2). TMCBR2 and TMCBR3 are both members of the *Kronosvirus pomeria* species [26] since they have 96.1% genome nucleotide identity. Similarly, W2 represents a second species designated *Kronosvirus elgin* [26]. Finally, Kronos represents a third species designated *Kronosvirus pelion* [26] since it has less than 95% genome nucleotide identity in pairwise comparisons with the other four related phage genomes. The phylogenetic relationship of these phage genomes is depicted in Figure 2.

**Figure 3:**
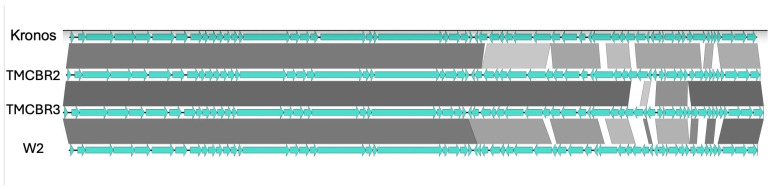
An alignment of four *Kronosvirus* genomes. The darkest color represents greater than 95% nucleotide identity. Each shade lighter represents a reduction of 5% nucleotide identity.

An alignment of the four *Kronosvirus* genomes revealed that the first 25 kb which contains the genes coding for the phage structural proteins is highly conserved (Figure 3). In addition, the interspecies comparisons showed much more genetic variation than observed with the within-species comparisons. A more detailed comparison of the related TMCBR2 and TMCBR3 *Kronosvirus pomeria* genomes revealed 388 single base changes and 75 indels (insertions or deletions) in a 10,000 bp region that corresponds to positions 28,750 to 38,750 in the TMCBR2 genome. In contrast, there are only 12 single base changes and one indel in the remaining 32 kb of the two genomes. Thus, this 10 KB region constitutes a hypervariable region relative to the remainder of these highly conserved phage genomes. Also, within the hypervariable region, the TMCBR2 genome contains one gene, gp045 (WCS66530.1), that is not present in the TMCBR3 genome. However, it is present in the Kronos genome (65% amino acid identity) suggesting that this gene has been deleted from the TMCBR3 genome. On the other hand, this region of the TMCBR3 genome contains two genes, gp051 (WDS38299.1) and gp052 (WDS38300.1), that are not present in the TMCBR2 genome or in any other genome that is present in the GenBank database based on an amino acid sequence BLAST (version 2.17.0) search. Thus, we conclude that these gene differences probably represent insertions of genes from unknown sources. Finally, the TMCBR2 genome is missing 10 bases that are present in the other four phage genomes, suggesting that these bases were deleted from the TMCBR2 genome.

The interspecies comparisons also revealed both nucleotide variation among shared genes and species-specific genes that were not present in the other species. For example, the W2 *Kronosvirus elgin* genome contained two genes, gp43 (WDS38411.1) and gp52 (WDS38420.1), that were different from the genes in the corresponding places in the other three genomes, and neither of these replacement genes matched any other genes that were present in the NCBI database. Similarly, the Kronos genome was missing a gene that is present in the other four genomes at approximately base pair 29,500 (gp034c WDS38403.1 W2; WCS66519.1 TMCBR2; WDS38282.1 TMCBR3). Interestingly, the TMCBR2 and TMCBR3 genes are identical, but different from the corresponding gene in the W2 genome. Finally, all three species had three species-specific genes in a 1200 bp region of the genomes that includes base pairs 38,800 through 40,000. Thus, based on our limited sampling of five phage genomes, the core genome of the *Kronosvirus* genus consists of 54 shared genes.

### 3.3. Evolutionary History

To identify potential relatives of the phages described in this study, we compared the predicted amino acid sequences of the large terminase subunit, the major capsid, and the tail tape measure genes to the GenBank ClusteredNR database using BLAST. Interestingly, we found no closely related amino acid sequences, and all the distantly related sequences (<60% amino acid identity) that we identified were present in the genomes of *Alphaproteobacteria*. These results indicate that (1) no phage genomes of relatives of these Kronos-related phages have been deposited in GenBank and that (2) the Kronos-related phages described in this paper are distantly related to a group of lysogenic phages that have integrated into Alphaproteobacterial genomes. Thus, it is possible that Kronos related phage particles could be produced by some of these lysogenic *Alphaproteobacteria*. However, the Kronos-related phage genes are not present in any of the 107 sequenced *Caulobacter* genomes. Thus, we conclude that the ability to establish lysogeny may have been lost before members of this group of phages began infecting members of the *Caulobacter* genus.

## 4. Discussion

We isolated three additional phages that infect *Caulobacter segnis* strain CBR1, and all three were shown to be members of the previously described *Kronosvirus* genus. However, the genetic diversity of these phages indicated that they should be classified into three separate species of the *Kronosvirus* genus based on the ICTV committee’s cutoff of 95% genome nucleotide sequence identity [26]. This level of genetic diversity probably reflects the natural genetic diversity of these phages since they were isolated from water samples obtained from geographically separate locations. Furthermore, we found no evidence of genes moving from one species lineage to another. This result is consistent with our previous observations that although the genomes of the members of the four genera in the *Dolichocephalovirinae* subfamily had lost or acquired genes that were not present in the other genera, none of the 69 genes that were present in all four genera of this subfamily had been transferred from one genus to another [11]. Taken together, these results suggest the acquisition of new genes does not involve recombination between closely related phage genomes in these two unrelated families of phages.

The *Kronosvirus* genus is unique among bacteriophages. To date, no phage genome has been sequenced that has more than 60% amino acid identity to the amino acid sequences of the large terminase subunit, the major capsid, or the tail tape measure proteins. Thus, the phage genome sequences described in this paper have made a significant contribution to our understanding of bacteriophage diversity.

## 5. Conclusions

The three new bacteriophages are closely related to Kronos and should be considered members of the *Kronosvirus* genus. However, pairwise whole genome comparisons showed that the four phages should be grouped into three distinct species. A detailed analysis of the nucleotide sequence variation demonstrated that most of the observed variation occurs in a portion of the genome that does not code for any known viral structural proteins. In addition, we have shown that within species and between species variation occurs both by single base differences in shared genes and by the addition or loss of whole genes. Only a few events that resulted in the gain or loss of genes were observed among the genomes of the three species indicating that the gene content of these genomes is relatively stable over an evolutionary time scale that allowed the accumulation of single base differences in numbers sufficient for speciation to occur.

## Figures and Tables

**Table 1:** Characteristics of the *Kronosvirus* genomes.

Phage Name NCBI Accession #	Kronos MH884648	TMCBR2 OQ269668	TMCBR3 OQ330849	W2 OQ330851
Genome size (bp)	42,244	42,838	43,231	42,284
Genomic GC content (%)	66.1	66.1	66.1	66.2
Total genes	58	60	62	59

**Table 2:** Results from a VIRIDIC comparison of the TMCBR2, TMCBR3, Kronos, TMCBR4, and W2 genomes. Numbers indicate the percent genome nucleotide identity.

	TMCBR2	TMCBR3	Kronos	W2
TMCBR2	100	96.1	89.1	89.9
TMCBR3		100	87.7	88.1
Kronos			100	91.5
W2				100

## Data Availability

The original contributions presented in this study are included in the article/Supplementary Materials. Further inquiries can be directed to the corresponding author.

## Supplementary Materials

The following supporting information can be downloaded at: https://www.mdpi.com/article/10.3390/v18040437/s1, Table S1: *Kronosvirus* gene comparisons.
