# Supplementary material for: New Species of Kronosvirus Bacteriophages That Infect Caulobacter Strain CBR1
Source: Viruses. 2026 Apr 5;18(4):437. doi: 10.3390/v18040437 (PMC13120211; doi:10.3390/v18040437)
Supplement: Supplementary file 1 [file viruses-18-00437-s001.zip › viruses-4179817-supplementary.pdf]

Supplementary Data: *Kronosvirus* gene comparisons

Kronos gene coordinates	*TMCBR2* OQ269668	*TMCBR3* OQ330849	*W2* OQ330851	*Gene product*
80..319	80-319	80-319	80-319	HNH endonuclease
540..1028	530-1018	540-1028	540-1028	Terminase, small subunit (TerS)
1032..2756	1022-2746	1032-2756	1032-2756	Terminase, large subunit (TerL)
2791..4083	2748-4073	2758-4083	2758-4083	Portal protein
4076..5038	4066-5028	4076-5038	4076-5038	Protease IV
5125..6489	5115-6479	5125-6489	5125-6489	Major capsid protein
6575..7309	6566-7312	6576-7322	6575-7309	Hypothetical protein
7480..8211	7480-8211	7490-8221	7480-8211	Gp6-like head-tail connector
8216..8596	8216-8596	8226-8606	8216-8596	Head closure Hc1
8600..9094	8600-9094	8610-9104	8600-9094	Tail completion
9094..9510	9094-9510	9104-9520	9094-9510	Tail terminator
9529..9987	9529-9987	9539-9997	9529-9987	Tail protein
9991..10410	9991-10410	10001-10420	9991-10410	Hypothetical protein
10434..10724	10434-10724	10444-10734	10434-10724	Hypothetical protein
10729..13623	10729-13620	10739-13630	10729-13623	Tail length-tape measure protein
13633..14220	13630-14217	13640-14227	13633-14220	Distal tail protein
14220..14864	14218-14862	14228-14872	14220-14864	Tail protein
14870..15358	14868-15356	14878-15366	14870-15358	Hypothetical protein
15355..18321	15347-18319	15363-18329	15349-18321	Tail fiber protein
18318..18746	18316-18744	18326-18754	18318-18746	Tail assembly chaperone protein
18756..19031	18754-19029	18764-19039	18756-19031	Hypothetical protein
19047..23066	19045-23064	19055-23074	19047-23066	Tail fiber protein
23069..23416	23067-23414	23077-23424	23069-23416	Hypothetical protein
23413..24144	23411-24142	23421-24152	23413-24144	Baseplate hub subunit and tail lysozyme
24148..24648	24146-24646	24156-24656	24148-24645	Hypothetical protein
24698..24874	24651-24875	24661-24885	24650-24874	Hypothetical protein
24983..25195c	24982-25191c	24992-25201c	24985-25196c	Hypothetical protein
25206..25772c	25201-25647c	25211-25657c	25207-25652c	Hypothetical protein
25873..26511c	25748-26386c	25758-26396c	25754-26391c	Hypothetical protein
26508..26813c	26383-26706c	26393-26716c	26389-26711c	Hypothetical protein
26828..27328c	26703-27203c	26713-27213c	26709-27208c	Hypothetical protein
27328..28521c	27203-28393c	27213-28403c	27209-28398c	Hypothetical protein
28590..29693c	28464-29570c	28474-29580c	28470-29578c	Hypothetical protein
Not present	29570-30034c	29580-30044c	29579-29901c	Hypothetical protein
29697..30266c	30038-30661c	30048-30671c	29905-30470c	Hypothetical protein
30344..31309c	30685-31650c	30695-31660c	30549-31513c	Hypothetical protein
31394..31822c	31735-32163c	31745-32272c	31599-32028c	DUF2303 protein
31983..32198c	32322-32537c	32333-32548c	32187-32401c	Hypothetical protein
32195..33403c	32534-33742c	32545-33753c	32399-33606c	Hypothetical protein
33407..33913c	33746-34255c	33757-34266c	33611-34125c	Hypothetical protein
33910..34281c	34252-34653c	34263-34640c	34123-34523c	Hypothetical protein
34278..34880c	34650-35222c	34637-35197c	34521-35092c	Hypothetical protein
34883..35530c	35225-35872c	35200-35790c	35096-35577c	Hypothetical protein
35543..35764c	35884-36105c	35803-36024c	35591-35811c	Hypothetical protein
35826..36095c	36167-36436c	36021-36536c	35874-36142c	Hypothetical protein
36147..36590	36587-36925	36686-37024	36162-36637	Hypothetical protein

Supplementary Data: *Kronosvirus* gene comparisons

36593..36748	36927-37082	37026-37181	36641-36795	Hypothetical protein
36745..37392	37079-37726	37178-37825	36793-37439	VRR-NUC domain-containing protein
37389..37784	37723-38121	37822-38286	37437-37831	Hypothetical protein
37784..38038	38121-38375	38286-38531	37832-38085	Hypothetical protein
Not present	Not present	38528-38761	Not present	Hypothetical protein
Not present	Not present	38758-38892	Not present	Hypothetical protein
38086..38475	38468-38812	38876-39205	38134-38522	HNH endonuclease
38472..39305	38809-39372	39202-39765	38520-39025	Hypothetical protein
39314..39604	39372-39674	39765-40067	39027-39328	Hypothetical protein
39601..39771	39671-39907	40064-40300	39326-39558	Hypothetical protein
Not present	39904-40101	40297-40494	Not present	Hypothetical protein
39768..39971	40098-40385	40491-40778	39556-39830	Hypothetical protein
39968..40222	40382-40636	40775-41029	39828-40081	Hypothetical protein
40267..41043	40681-41457	41074-41850	40127-40902	DNA methyltransferase
41040..41774	41454-42188	41847-42581	40900-41633	Replication initiation O-like
41809..42390	42223-42804	42616-43197	41669-42249	Hypothetical protein
